# SaMpling Antibiotics in Renal Replacement Therapy (SMARRT): an observational pharmacokinetic study in critically ill patients

**DOI:** 10.1186/s12879-016-1421-6

**Published:** 2016-03-01

**Authors:** Jason A. Roberts, Gordon Y. S. Choi, Gavin M. Joynt, Sanjoy K. Paul, Renae Deans, Sandra Peake, Louise Cole, Dianne Stephens, Rinaldo Bellomo, John Turnidge, Steven C. Wallis, Michael S. Roberts, Darren M. Roberts, Melissa Lassig-Smith, Therese Starr, Jeffrey Lipman

**Affiliations:** Burns Trauma and Critical Care Research Centre, The University of Queensland, Level 3 Ned Hanlon Building, Royal Brisbane and Women’s Hospital, Herston, Queensland, 4029 Australia; Royal Brisbane & Women’s Hospital, Queensland, Australia; Prince of Wales Hospital, The Chinese University of Hong Kong, Hong Kong, Special Administrative Region China; Clinical Trials & Biostatistics Unit, QIMR Berghofer, Queensland, Australia; The Queen Elizabeth Hospital, South Australia, Australia; Nepean Hospital, New South Wales, Australia; Royal Darwin Hospital, Northern Territory, Australia; Austin Hospital, Victoria, Australia; Royal Women’s and Children’s Hospital, Queensland, Australia; Therapeutics Research Unit, The University of Queensland, Queensland, Australia

**Keywords:** Sepsis, Antibiotic dosing, Dialysis, Pharmacokinetics, Pharmacodynamics, Vancomycin, Piperacillin, Tazobactam, Meropenem, Linezolid

## Abstract

**Background:**

Optimal antibiotic dosing is key to maximising patient survival, and minimising the emergence of bacterial resistance. Evidence-based antibiotic dosing guidelines for critically ill patients receiving RRT are currently not available, as RRT techniques and settings vary greatly between ICUs and even individual patients. We aim to develop a robust, evidence-based antibiotic dosing guideline for critically ill patients receiving various forms of RRT. We further aim to observe whether therapeutic antibiotic concentrations are associated with reduced 28-day mortality.

**Methods/Design:**

We designed a multi-national, observational pharmacokinetic study in critically ill patients requiring RRT. The study antibiotics will be vancomycin, linezolid, piperacillin/tazobactam and meropenem. Pharmacokinetic sampling of each patient’s blood, RRT effluent and urine will take place during two separate dosing intervals. In addition, a comprehensive data set, which includes the patients’ demographic and clinical parameters, as well as modality, technique and settings of RRT, will be collected. Pharmacokinetic data will be analysed using a population pharmacokinetic approach to identify covariates associated with changes in pharmacokinetic parameters in critically ill patients with AKI who are undergoing RRT for the five commonly prescribed antibiotics.

**Discussion:**

Using the comprehensive data set collected, the pharmacokinetic profile of the five antibiotics will be constructed, including identification of RRT and other factors indicative of the need for altered antibiotic dosing requirements. This will enable us to develop a dosing guideline for each individual antibiotic that is likely to be relevant to any critically ill patient with acute kidney injury receiving any of the included forms of RRT.

**Trial registration:**

Australian New Zealand Clinical Trial Registry (ACTRN12613000241730) registered 28 February 2013

## Background

Acute kidney injury (AKI) is a common complication of critical illness, and patients requiring renal replacement therapy (RRT) have high hospital mortality rates [1, 2]. The cause of AKI is often multifactorial, however, sepsis and septic shock remain the most important causes of AKI in the critically ill, and account for over 50 % of cases of AKI. Compared with non-septic AKI, septic AKI is associated with greater derangements in haemodynamic and laboratory parameters, greater severity of illness, and higher need for mechanical ventilation and vasoactive therapy [3]. Despite adjustment for covariates, when compared with non-septic AKI, patients with septic AKI have a 50 % higher risk of death, and survivors require a long duration of hospitalisation [4].

To maximise patient survival, early recognition of sepsis, and the timely application of antibiotics against the offending pathogen(s) is required [5]. Therapeutic antibiotic concentrations need to be attained rapidly and then appropriately maintained in order to maximise bacterial killing [6–8]. Achieving therapeutic concentrations in blood may also serve to minimise toxicity and reduce the emergence of antibiotic resistance [9, 10]. Optimisation of antibiotic dosing to achieve defined pharmacokinetic (PK)/pharmacodynamic (PD; PK/PD) targets, has been proposed as one such therapeutic approach [11, 12]. Existing antibiotic dosing regimens are designed to achieve PK/PD targets, but assume the PK of a non-critically ill patient, and are often poorly validated in the intensive care unit (ICU) setting. There is a growing body of evidence demonstrating the existence of significant changes in antibiotic PK in critically patients, particularly those with sepsis and septic shock [13–15].

When sepsis induces AKI of sufficient severity, RRT is prescribed to remove waste solutes by convection, known as haemofiltration (HF); diffusion, known as haemodialysis (HD); or a combination of the two, known as haemodiafiltration (HDF). To date, there is limited evidence demonstrating a clinical outcome advantage for any one form of RRT over another. Therefore, there is significant heterogeneity in the clinician’s preference and use of the various RRT modalities and settings. In clinical use, HD will clear predominantly small molecules (e.g. urea, creatinine and small antibiotics), whereas HF also allows the additional clearance of much larger molecules [16]. The application of RRT significantly complicates antibiotic dosing with factors such as altered protein binding and antibiotic hydrophilicity affecting PK. In addition, the operational characteristics of RRT, such as mode, filter type, blood flow rate, membrane fouling, fluid replacement site and total effluent rate, all can influence drug disposition [17].

Changes in antibiotic PK are best described in terms of changes to the primary PK parameters, volume of distribution (Vd) and clearance. At its most basic level, Vd is the PK parameter that determines antibiotic loading dose requirements. Hydrophilic antibiotics are usually confined to the intravascular and interstitial fluid of tissues and have a lower Vd than more lipophilic antibiotics (like many quinolones). In the acute phase of critical illness, fluid resuscitation coupled with reduced protein binding can increase the Vd. An increased Vd has been demonstrated for penicillins [18], carbapenems [19] and glycopeptides [20]. Where critical illness causes a larger Vd for an antibiotic, lower antibiotic concentrations will result if the dosage is not adjusted [20, 21], supporting the need for higher doses in the acute phase of treatment. Linezolid has a larger Vd than these other antibiotics and this is not considered to change significantly during critical illness [22].

The maintenance dose of antibiotic is primarily determined by clearance (CL). For antibiotics such as vancomycin, piperacillin/tazobactam and meropenem, CL is predominantly by the kidneys [23–25]. In patients with AKI, RRT is used to substitute the loss of renal function. The modalities that may be selected by the physician include continuous veno-venous haemofiltration (CVVHF), continuous veno-venous haemodiafiltration (CVVHDF), intermittent haemodialysis (IHD) and extended daily diafiltration (EDD-f) (EDD-f is an 8–12 per 24 h RRT treatment using a modified IHD setup with the addition of an online filtration component).

Although the effect of different RRT settings on antibiotic PK and blood concentrations has been previously described in several small studies, data quantifying the interaction of altered PK and RRT covariates remains limited [26]. One meta-review by Jamal et al. examined the effect of RRT settings on meropenem, piperacillin and vancomycin CL. The authors found that the setting most correlated with antibiotic CL was effluent flow rate for meropenem (Spearman correlation coefficient (rs) = 0.43; *p* = 0.12), piperacillin (rs = 0.77; *p* = 0.10), and vancomycin (rs = 0.90; *p* = 0.08). It could perhaps be concluded that maintenance dosing of these antibiotics could be guided by the effluent flow rate. However, in a recent multicentre pharmacokinetic study, RRT dosage (effluent rate) itself was not sufficient to be a sole predictor of trough concentrations, and a greater understanding of other factors that may guide dosing [27]. We therefore propose that suboptimal antibiotic dosing likely occurs during RRT.

Our study will address the above knowledge deficiency. We aim to develop an individualised optimal dosing algorithm that accounts for the relevant effects of RRT modalities and patient clinical characteristics on dosing requirements for vancomycin, linezolid, piperacillin-tazobactam and meropenem. This knowledge will significantly improve antibiotic dosing by providing robust dosing algorithms for critically ill patients receiving RRT. The findings will have worldwide relevance and applicability.

## Methods

The study is endorsed by the Australian and New Zealand Intensive Care Society Clinical Trials Group (ANZICS-CTG) and has been registered with the Australian New Zealand Clinical Trial Registry (ACTRN12613000241730).

### Design

This is a multi-national observational PK study designed to analyse PK data using a population PK approach to identify covariates associated with changes in PK parameters for five commonly prescribed antibiotics, vancomycin, linezolid, piperacillin/tazobactam and meropenem in critically ill patients with AKI who are undergoing RRT.

### Participants and study sites

By using an international collaborative approach from over 30 participating ICUs, the study will enrol a minimum of 450 patients, who satisfy the protocol-defined inclusion and exclusion criteria in Table 1. Patients will be removed from the study should they meet the withdrawal criteria listed in Table 2.

**Table 1:** Inclusion and exclusion criteria

Inclusion criteria • Age 18 years or older • AKI requiring RRT (defined according to RIFLE^a^, AKIN^b^ or KDIGO^c^ criteria) • Expected to require RRT for at least 4 days with two (2) sampling sechedules • Clinical indication for IV piperacillin-tazobactam, meropenem, imipenem-cilastatin, vancomycin or linezolid • Presence of intra-arterial line for blood sampling if RRT filter port sampling not possible • Informed consent from patient or patient’s authorised representative Exclusion criteria • Imminent death/patient not expected to survive • Major bleeding or blood haemoglobin concentration <70 g/L or platelets <20 x 10^9^/L • Regular dosing with any of the 5 study antibiotics for greater than 36 h, within the 7 days prior to enrolment • Unable to obtain consent	

^a^Bellomo R *et al.* Crit Care 2004, 8:R204-R212 (DOI 10.1186/cc2872)

^b^Metha RL et al. Crit Care 2007, 11:R31 (doi:10.1186/cc5713)

^c^KidneyDisease: Improving Global Outcomes (KDIGO) Acute Kidney Injury Work Group. KDIGO Clinical Practice Guideline for Acute Kidney Injury. Kidney Int Suppl 2012, 2:1-138

Abbreviations: *AKI* acute kidney injury, *RRT* renal replacement therapy, *RIFLE* Risk, Injury, Failure, Loss and End stage Renal Disease, *AKIN* Acute Kidney Injury Network, *KDIGO* Kidney Disease Improving Global Outcomes, *IV* intravenous

**Table 2:** Withdrawal criteria for SMARRT study

• Major Bleeding • Blood haemoglobin concentration < 70 g/L • Platelets < 20 x 10^9^/L	

### Study treatments

The number of eligible patients to be enrolled for any individual antibiotic is 150 for patients receiving vancomycin, piperacillin-tazobactam, or meropenem. For linezolid, at least 50 patients will be enrolled. Each study antibiotic will be administered and dosed according to standard administration guidelines at each participating centre. The study will be conducted in centres with critically ill patients receiving CVVHF, CVVHDF or EDD-f. The RRT prescription choice will be determined by local clinical practice. It is likely that, given the variety and geographical distribution of the participating ICUs, that most common combinations of RRT prescription within each modality will be included in this study (Table 3). The pre-study survey information about the participating sites confirm that the study will provide different modes of RRT, with variations of filter surface area (>3-fold), blood flow rate (>2-fold) and effluent flow rate (>14-fold), which together will provide an excellent opportunity to analyse the effect of altered RRT settings and/or modalities on antibiotic PK.

**Table 3:** RRT Modalities and range of setting at participating sites

RRT Modality	Filter surface area (m^2^)	Blood flow rate (ml/min)	Effluent flow rate (ml//kg/h)
CVVHDF	0.9–1.05	180–200V	25–40
CVVHF	1.2–2.15	130–250	25–100
EDD-f	0.6–1.4	200–300	60–350

Abbreviations: *RRT* renal replacement therapy, *CVVHDF* continuous venovenous haemodiafiltration, *CVVHF* continuous venovenous haemofiltration, *EDD-f* extended daily dialfiltration

Each site aims to recruit at least of 8–10 patients per antibiotic. The sampling schedule (see section on PK Samples) is defined and will be conducted for each patient on two separate dosing intervals aiming to describe PK changes over the initial course of antibiotic therapy. Data on patient demographics, clinical parameters, RRT modality and operational characteristics will be collected and analysed in line with the paper from Li et al [28] (see Data Analysis).

### Pharmacokinetic samples

**Fig. 1:**
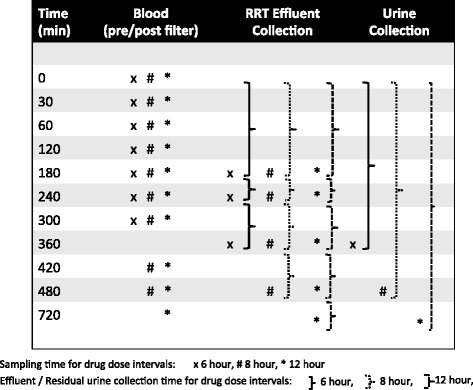
Antibiotic Pharmacokinetic Sampling Schedule

Study samples will be taken from each patient when possible during one dosing interval on days 1 or 2 and then again during another dosing interval on days 3–6 of commencing the study antibiotics. As described in Fig. 1, serial samples of plasma, effluent and urine (in patients with residual urine output) will be measured to allow construction of PK profiles.

### Plasma samples

Plasma samples will be collected pre- and post filter from designated ports within the RRT circuit at the following time points (0, 30, 60, 120, 180, 240, 300, 360, 420 and 480 min), where time zero (0) denotes the baseline sampling time prior to the administration of the study antibiotic. If drugs are administered over 12 h, a 720 min sample will also be collected. Each sample will consist of 3 ml of blood (total blood removed per day is 60 ml; 66 ml if 12 h antibiotic dosing is used). All the collected blood samples will be centrifuged at 3000 rpm and separated before storage. If the filter port is not accessible for an individual time point, arterial line sampling will be performed for PK analysis.

### Urine samples

Urine produced during the sample collection period will be collected (if available) and will be used to calculate residual renal function based on the rate of creatinine clearance. Urine samples will be measured for creatinine concentrations by the local pathology laboratory servicing individual ICUs in line with standard procedures. This typically involves collection of the entire volume of urine, and creatinine clearance calculated based on the formula:

$$ \mathrm{U}\mathrm{c}\mathrm{r}\ \mathrm{x}\ \mathrm{V}\ /\ \mathrm{P}\mathrm{c}\mathrm{r} $$

Where,

Ucr = Urine concentration of creatinine

Pcr = Plasma concentration of creatinine

V = volume of urine per unit time

Before the residual urine is sent to the local pathology laboratory, a one ml urine aliquot will be transferred to a urine collection jar and labelled for storage and later analysis of antibiotic concentration.

### Effluent samples

An empty RRT effluent bag will be inserted at the start of the dosing interval. The RRT filter will not be routinely changed at the start of sampling and the age of the filter will be recorded. New effluent bag(s) will be used during each sampling time period: 0–180 min, 180–240 min, 240–360 min, 360–480 min, and 480–720 min (for 12-h dosing). An aliquot (1 ml) of effluent will be taken from the effluent bag at the time of changing, at time points 180, 240, 360 and 480 min (and 720 min if 12 h dosing). If more than one effluent bag is required during the effluent sampling period, then 1 ml of effluent shall be taken from each effluent bag used, up to a total of 5 samples (i.e. 1 ml from each of the 5 effluent bags). Throughout the sampling period, the amount of antibiotic cleared by RRT will be determined by recording the volume and concentration of effluent.

Analysis of effluent antibiotic concentration collected at the following time points – 180, 240, 360 and 480 min (and 720 min if 12 hourly dosing) – will enable the calculation of the sieving coefficient (Sc) or saturation coefficient for dialysate (Sd) [17]. Drug clearance by RRT will be described by calculation of Sc for CVVHF or Sd for CVVHDF using the following equation:

$$ \mathrm{S}\mathrm{c}\ \left(\mathrm{or}\ \mathrm{S}\mathrm{d}\right) = \left(\mathrm{Concentration}\ \mathrm{of}\ \mathrm{d}\mathrm{rug}\ \mathrm{in}\ \mathrm{effluent}\right)/\left(\left(\left[\mathrm{Plasma}\ \mathrm{c}\mathrm{oncentration}\ \mathrm{of}\ \mathrm{d}\mathrm{rug}\right]\ \mathrm{prefilter} + \left[\mathrm{Plasma}\ \mathrm{c}\mathrm{oncentration}\ \mathrm{of}\ \mathrm{d}\mathrm{rug}\right]\ \mathrm{postfilter}\right)/2\right) $$

Plasma, RRT effluent and urine will be stored at -70 °C to -80 °C. Samples will be stored at individual sites awaiting bulk transport by an international courier to the Burns, Trauma, & Critical Care Research Centre, The University of Queensland, Brisbane, Queensland, Australia for assay by validated chromatographic methods. An example of the PK sampling schedule for the different dosing regimens is outlined in Fig. 1.

#### Ethics

The study has received ethics approval from the Royal Brisbane and Women’s Hospital Human Research Ethics Committee (HREC/13/QRBW/1) as the lead site. All other participating sites have also obtained individual HREC approval.

#### Data collection and safety monitoring

Trained staff at each study centre will collect data and information will be entered into a web-based clinical trial database system (OpenClinica, LLC). Information to be collected on the case report form is outlined in Table 4. Personnel trained by the coordinating centre at The University of Queensland will conduct monitoring for the study. The first monitoring visit will be scheduled after the participating centre has enrolled at least one patient, with 100 % source data verification, which includes monitoring of participant consent, sample collection timesheet, adverse events, protocol deviation and vital status at day 28 as secondary outcome. Monitoring for subsequent patients will be performed through source data verification of the case report form and review of the OpenClinica patient profile.

**Table 4:** Parameters recorded in the study case report form

Admission details • Patient characteristics (identification number, age, sex) • Inclusion and exclusion criteria • Consent details • Co-enrolment • Date of hospital admission • Patient’s height and weight • Admission diagnosis • Date of ICU admission • APACHE II score on ICU admission Clinical details • Site of infection • Identification of pathogen(s) and susceptibility to study antibiotic • SOFA score (ICU admission, and sampling occasions 1 and 2) • Cumulative fluid balance • Medication details (antibiotic(s), dose administered, dosing intervals) • Clinical response Renal Replacement Therapy • Modality • Filter membrane size • Filter membrane material • Transmembrane pressure • Replacement fluid flow rate (pre/post dilution) • Dialysate fluid flow rate • Targeted hourly fluid removal rate Laboratory Investigations • Blood sampling time • Measured effluent volume at pre-defined collection time • Effluent sampling time • Residual measured urinary creatinine clearance • Residual urine sampling time • Haematocrit • Liver function tests Completion and outcome details • Reason for discontinuation/withdrawal • Reason for protocol deviation/violation • Adverse events • Vital status at ICU and hospital discharge • 28 day survival	

Abbreviations: *ICU* Intensive care unit, *APACHE* Acute Physiology and Chronic Health Evaluation, *SOFA* Sequential Organ Failure Assessment Score

Given the observational nature of the study, major adverse events attributed to the study are not anticipated. Participating centres will report all adverse events up to day 28 in which study participation was a contributing factor to the coordinating centre. Should an adverse event occur, the coordinating centre will be responsible for providing appropriate patient follow up, and ensure all details are recorded for the study management committee to review. All serious adverse events will be reported to the Royal Brisbane and Women’s Hospital Human Research Ethics Committee and to local institutional ethics committees or jurisdictional bodies.

## Statistical design and analysis approach

The study aims to develop a dosing guideline of global significance for the commonly used antibiotics in ICU patients [29–32]. This process will be informed by a better understanding of the distribution of relevant PK parameters and the extent they are influenced by RRT modalities and settings, and individual patient characteristics.

### Population pharmacokinetic analysis

The concentration-time data for each antibiotic in plasma, RRT effluent and urine will be fitted using nonlinear mixed-effects modeling (NONMEM version 7.3, Globomax LLC, Hanover, USA) [33]. A Digital Fortran compiler will be used and the runs will be executed using Wings for NONMEM (http://wfn.sourceforge.net). Data will be analyzed using the first-order conditional estimation method with interaction. We will attempt to include between-subject variability and between occasion variability using exponential variability models and residual unexplained variability using a combined exponential and/or additive random error model as appropriate. Goodness of fit will be evaluated by visual inspection of diagnostic scatter plots and evaluation of the NONMEM objective function value (OFV). Statistical comparison of nested models will then be undertaken in the NONMEM program on the basis of a Chi square (χ^2^) test of the difference in OFV. A decrease in the OFV of 3.84 units (*P* < 0.05) will be considered statistically significant. The final model will be evaluated by performing a visual predictive check (VPC), and by evaluation of other goodness of fit plots.

### Statistical analysis

Using the population PK parameter estimates, a robust preliminary dosing algorithm will then be developed through the following design-based sequential analytical approaches:

- Determination of the effect of various patient and RRT factors on PK parameters: this analysis will provide the effect size and significance of various patient and RRT factors on the confidence boundaries of individual PK parameters obtained from basic models. This approach will provide robust estimates of differences in average levels of individual PK parameters by RRT modalities.
- Evaluation of statistical distributions of PK parameters and determination of the optimum antibiotic dosing limits in patients receiving different RRT modes: this analysis will explore appropriate models to determine the confidence boundaries of PK parameters, CL and Vd for each antibiotic during the different RRT modes and settings, and will then use a simulation approach using these basic models to define the range of appropriate doses for the antibiotics.
- Development of an enhanced preliminary prediction algorithm for antibiotic dosing: this final step will incorporate the statistically significant and clinically relevant predictors from step (a) into the basic models from step (b) to attempt to develop a robust dosing guideline for individual forms of RRT.

### Sample size and power

The power analysis is aimed at establishing the confidence boundaries of individual PK parameters under different RRT modalities and settings. For vancomycin, piperacillin/tazobactam and meropenem, to construct a 95 % confidence boundary of clearance, with standard deviation as high as 2.5 L/h with 80 % power, we will need 45 samples of patient data for each particular RRT modality. For the three modalities for these antibiotics, we will need a minimum of 135 patients per antibiotic. A total sample of 450 patients for the three antibiotics (150 patients each) is required to allow for potential attrition. Based on pre-study survey activities linezolid is prescribed less frequently at the study sites. Therefore, we will construct a 95 % confidence boundary of clearance, with standard deviation as high as 1.5 L/h which requires 15 patients per antibiotic per RRT modality (45 patients per antibiotic). We will enrol at least 50 patients for linezolid to allow for potential attrition.

## Discussion

The difficulty in predicting the PK profile of ICU patients receiving RRT lies in the physiological alterations of critical illness and the different operational characteristics of RRT [17]. This, in turn, challenges an optimised approach to antibiotic prescribing for these patients, with failure to achieve optimal blood antibiotic concentrations and potentially a negative impact on survival [1, 2]. The aim of the study is to quantify the effects of critical illness and the use of RRT on the PK parameters of five common antibiotics in a large cohort of ICU patients and to attempt to develop robust antibiotic dosing guidelines for ICU patients that account for specific patient characteristics and the type of RRT they are prescribed.

The investigators hypothesise that the uncertainty of the magnitude of the influence of patient and RRT factors on PK can be defined by the systematic exploration of these factors in a large, multi-centre ICU patient cohort, such as that to be investigated in this study. The first step is the recording of the detailed description of enrolled patients’ demographic and clinical parameters, particularly organ function (defined by components of the Sequential Organ Failure Assessment (SOFA) score), RRT modalities and settings, together with the plasma antibiotic concentration-time data. This comprehensive data set will permit the description of detailed PK data, and allow further analysis of the effect of critical illness, AKI and RRT on the drug disposition of five of the most commonly used antibiotics in ICU patients, namely, vancomycin, linezolid, piperacillin/tazobactam and meropenem.

Following this, it will be possible to evaluate the statistical distributions of PK parameters and determine the optimum antibiotic dosing limits during different regimens of RRT. This analysis will explore appropriate models to determine the confidence boundaries of PK parameters, such as Vd and CL for each antibiotic during the different regimens of RRT, adjusted for relevant patient characteristics. The information obtained will then be used, via a simulation approach, in these basic models to define the range of appropriate doses for the studied antibiotics.

Finally, analysing the effect of various patient and RRT factors on PK parameters should provide an estimate of the effect size and significance of various patient and RRT factors on the confidence boundaries of individual PK parameters obtained from the basic models derived earlier. This approach should provide robust estimates of changes in individual PK parameters induced by critical illness and by different RRT modalities. In essence, population PK modeling should allow us to quantify the levels of variability shown in these patients, and the effect of each individual physiological and pathological characteristic. In a similar way, the statistical modeling should allow the quantification of the effects of different modes and settings of RRT on the disposition of the studied antibiotics.

Utilising the information developed through the above process, we expect to be able to develop an enhanced preliminary prediction algorithm for antibiotic dosing. This final step will incorporate the statistically significant and clinically relevant predictors from the above steps, into initial basic models, thereby developing a robust dosing guideline for commonly used forms of RRT. If such dosing guidelines are successful, they are likely to improve the effectiveness of the prescribed therapy against target pathogens by ensuring clinically effective blood concentrations of the antibiotics investigated. The enhanced preliminary prediction algorithm may then undergo subsequent validation by means of a large-scale randomised control trial with mortality as a primary outcome.

## Conclusions

Current guidelines for dosing in septic critically ill patients with AKI receiving RRT are likely to be inadequate. The modality and operational settings of RRT can be significantly different between different ICUs. These different practices result in meaningfully different antibiotic blood concentrations and as such a singular antibiotic dose to cover all RRT scenarios is unlikely to be effective. This study is the first multi-national observational PK study aimed at determining the important factors that contribute to antibiotic blood concentrations, attempting to generate models to predict these changes and to utilise the models to improve clinical guidelines. To achieve this, the PK variability of the five antibiotics commonly used in patients with AKI who are undergoing RRT will be investigated. Robust data from this multi-centre cohort of patients should provide the required PK knowledge with sufficient statistical power to develop an enhanced algorithm for antibiotic dosing in this specific population of critically patients.

## Abbreviations

ACTRN: Australian New Zealand Clinical Trial Registry
ANZICS-CTG: Australian and New Zealand Intensive Care Society Clinical Trials Group
AKI: Acute Kidney Injury
CL: Clearance
CVVHDF: Continuous veno-venous haemodiafiltration
CVVHF: Continuous veno-venous haemofiltration
EDD-f: Extended daily diafiltration
HD: Haemodialysis
HDF: Haemodiafiltration
HF: Haemofiltration
HREC: Human Research Ethics Committee
ICU: Intensive care unit
IHD: Intermittent haemodialysis
NONMEM: Nonlinear mixed effects modeling
OFV: Objective Function Value
Pcr: Plasma concentration of creatinine
PD: Pharmacodynamics
PK: Pharmacokinetics
RRT: Renal Replacement Therapy
Sc: Sieving coefficient
Sd: Saturation coefficient
SOFA: Sequential Organ Failure Assessment
Ucr: Urine concentration of creatinine
V: Volume of urine
Vd: Volume of distribution
VPC: Visual predictive check
χ^2^: Chi square

## References

[1] Bagshaw SM (2006). The long-term outcome after acute renal failure. Curr Opin Crit Care.

[2] Ahlström A, Tallgren M, Peltonen S, Räsänen P, Pettilä V (2005). Survival and quality of life of patients requiring acute renal replacement therapy. Intensive Care Med.

[3] Uchino S, Kellum JA, Bellomo R, Doig GS, Morimatsu H, Morgera S (2005). Acute renal failure in critically ill patients: a multinational, multicenter study. JAMA..

[4] Bagshaw SM, Uchino S, Bellomo R, Morimatsu H, Morgera S, Schetz M (2007). Septic acute kidney injury in critically ill patients: clinical characteristics and outcomes. Clin J Am Soc Nephrol..

[5] Kumar A, Roberts D, Wood KE, Light B, Parrillo JE, Sharma S (2006). Duration of hypotension before initiation of effective antimicrobial therapy is the critical determinant of survival in human septic shock. Crit Care Med.

[6] Roberts JA, Paul SK, Akova M, Bassetti M, De Waele JJ, Dimopoulos G (2014). DALI: defining antibiotic levels in intensive care unit patients: are current β-lactam antibiotic doses sufficient for critically ill patients?. Clin Infect Dis.

[7] Moise-Broder PA, Forrest A, Birmingham MC, Schentag JJ (2004). Pharmacodynamics of vancomycin and other antimicrobials in patients with Staphylococcus aureus lower respiratory tract infections. Clin Pharmacokinet.

[8] Rayner CR, Forrest A, Meagher AK, Birmingham MC, Schentag JJ (2003). Clinical pharmacodynamics of linezolid in seriously ill patients treated in a compassionate use programme. Clin Pharmacokinet.

[9] Roberts JA, Norris R, Paterson DL, Martin JH (2012). Therapeutic drug monitoring of antimicrobials. Br J Clin Pharmacol.

[10] Roberts JA, Kruger P, Paterson DL, Lipman J (2008). Antibiotic resistance--what’s dosing got to do with it?. Crit Care Med.

[11] De Paepe P, Belpaire FM, Buylaert WA (2002). Pharmacokinetic and pharmacodynamic considerations when treating patients with sepsis and septic shock. Clin Pharmacokinet.

[12] Roberts JA, Abdul-Aziz MH, Lipman J, Mouton JW, Vinks AA, Felton TW (2014). Individualised antibiotic dosing for patients who are critically ill: challenges and potential solutions. Lancet Infect Dis..

[13] Varghese JM, Roberts JA, Lipman J (2011). Antimicrobial pharmacokinetic and pharmacodynamic issues in the critically ill with severe sepsis and septic shock. Crit Care Clin.

[14] Udy AA, Roberts JA, Lipman J (2013). Clinical implications of antibiotic pharmacokinetic principles in the critically ill. Intensive Care Med.

[15] Roberts JA, Joynt GM, Choi GY, Gomersall CD, Lipman J (2012). How to optimise antimicrobial prescriptions in the Intensive Care Unit: principles of individualised dosing using pharmacokinetics and pharmacodynamics. Int J Antimicrob Agents.

[16] Bellomo R, Tipping P, Boyce N (1993). Continuous veno-venous hemofiltration with dialysis removes cytokines from the circulation of septic patients. Crit Care Med.

[17] Choi G, Gomersall CD, Tian Q, Joynt GM, Freebairn R, Lipman J (2009). Principles of antibacterial dosing in continuous renal replacement therapy. Crit Care Med.

[18] Varghese JM, Jarrett P, Boots RJ, Kirkpatrick CM, Lipman J, Roberts JA (2014). Pharmacokinetics of piperacillin and tazobactam in plasma and subcutaneous interstitial fluid in critically ill patients receiving continuous venovenous haemodiafiltration. Int J Antimicrob Agents.

[19] Isla A, Rodríguez-Gascón A, Trocóniz IF, Bueno L, Solinís MA, Maynar J (2008). Population pharmacokinetics of meropenem in critically ill patients undergoing continuous renal replacement therapy. Clin Pharmacokinet.

[20] DelDot ME, Lipman J, Tett SE (2004). Vancomycin pharmacokinetics in critically ill patients receiving continuous venovenous haemodiafiltration. Br J Clin Pharmacol.

[21] Seyler L, Cotton F, Taccone FS, De Backer D, Macours P, Vincent JL (2011). Recommended β-lactam regimens are inadequate in septic patients treated with continuous renal replacement therapy. Crit Care.

[22] Meyer B, Kornek GV, Nikfardjam M, Karth GD, Heinz G, Locker GJ (2005). Multiple-dose pharmacokinetics of linezolid during continuous venovenous haemofiltration. J Antimicrob Chemother.

[23] Moellering RC (1984). Pharmacokinetics of vancomycin. J Antimicrob Chemother.

[24] Sörgel F, Kinzig M (1993). The chemistry, pharmacokinetics and tissue distribution of piperacillin/tazobactam. J Antimicrob Chemother.

[25] Mouton JW, van den Anker JN (1995). Meropenem clinical pharmacokinetics. Clin Pharmacokinet.

[26] Jamal JA, Udy AA, Lipman J, Roberts JA (2014). The impact of variation in renal replacement therapy settings on piperacillin, meropenem, and vancomycin drug clearance in the critically ill: an analysis of published literature and dosing regimens*. Crit Care Med.

[27] Roberts DM, Roberts JA, Roberts MS, Liu X, Nair P, Cole L (2012). Variability of antibiotic concentrations in critically ill patients receiving continuous renal replacement therapy: a multicentre pharmacokinetic study. Crit Care Med..

[28] Li AM, Gomersall CD, Choi G, Tian Q, Joynt GM, Lipman J (2009). A systematic review of antibiotic dosing regimens for septic patients receiving continuous renal replacement therapy: do current studies supply sufficient data?. J Antimicrob Chemother.

[29] Erdem H, Inan A, Altındis S, Carevic B, Askarian M, Cottle L (2014). Surveillance, control and management of infections in intensive care units in Southern Europe, Turkey and Iran--a prospective multicenter point prevalence study. J Infect.

[30] Curcio D, Latin American Antibiotic Use in Intensive Care Unit Group† (2013). Antibiotic prescriptions in critically-ill patients: a latin american experience. Ann Med Health Sci Res.

[31] Fowler RA, Flavin KE, Barr J, Weinacker AB, Parsonnet J, Gould MK (2003). Variability in antibiotic prescribing patterns and outcomes in patients with clinically suspected ventilator-associated pneumonia. Chest.

[32] Rello J, Ulldemolins M, Lisboa T, Koulenti D, Mañez R, Martin-Loeches I (2011). Determinants of prescription and choice of empirical therapy for hospital-acquired and ventilator-associated pneumonia. Eur Respir J.

[33] Boeckmann AJ, Sheiner LB, Beal SL (1994). NONMEM Users Guide - Part V: Introductory Guide.

